# Serostatus‐Specific Prognostic Profiles and Phenotype‐Dependent Therapeutic Windows in Autoimmune Encephalitis: A Multicenter Cohort Study

**DOI:** 10.1002/acn3.70571

**Published:** 2026-10-28

**Authors:** Xiaohong Tang, Jingxian Wang, Kaixin Zhu, Zhen Zhang, Liao Wu, Wei Wang, Chang Li, Qingyun Li, Hao Li, Jinghua Chen, Zhangxu Lian, Bin Liu, Tengfei Liu, Chunmei Wen, Nuan Wang, Sen Qun, Lishun Xiao, Qingwei Lai

**Affiliations:** ^1^ Department of Neurology The Affiliated Hospital of Xuzhou Medical University Xuzhou China; ^2^ Department of Neurology People's Hospital of Hongze District Huaian China; ^3^ Department of Medical Records Nanjing Brain Hospital Nanjing China; ^4^ Department of Neurology The First Affiliated Hospital of University of Science and Technology of China Hefei China; ^5^ Department of Neurology Xuzhou Central Hospital Xuzhou China; ^6^ Department of Neurology The Affiliated Huai'an No. 1 People's Hospital of Nanjing Medical University Huai'an China; ^7^ Department of Neurology Yancheng First People's Hospital Yancheng China; ^8^ Department of Neurology The Fourth Affiliated Hospital of Soochow University Suzhou China; ^9^ Department of Oncology The First Affiliated Hospital of Shandong First Medical University Jinan China; ^10^ Department of Geriatric Medicine Nanjing Brain Hospital Nanjing China; ^11^ Department of Neurology Xuzhou Mining Group General Hospital Xuzhou China; ^12^ Department of Neurology Yancheng Third People's Hospital Yancheng China; ^13^ Department of Neurology First People's Hospital of Xuzhou Xuzhou China; ^14^ Division of Life Sciences and Medicine University of Science and Technology of China Hefei China; ^15^ School of Public Health Xuzhou Medical University Xuzhou China; ^16^ Department of Neurology, The First Clinical College Xuzhou Medical University Xuzhou China

**Keywords:** autoimmune encephalitis, mixed‐effects model, prognosis, risk stratification, seronegative

## Abstract

**Background:**

While autoimmune encephalitis (AE) outcomes vary, serostatus‐specific prognostic determinants and institutional clustering effects remain poorly characterized. We aimed to delineate the distinct prognostic architecture of AE, identifying serostatus‐specific risk factors and potential therapeutic windows.

**Methods:**

This multicenter, retrospective cohort study (2016–2024) included 498 patients (382 antibody‐positive [Ab+], 116 antibody‐negative [Ab−]) across 11 tertiary centers. The primary endpoint was 1‐year poor functional outcome (mRS > 2). We utilized Least Absolute Shrinkage and Selection Operator (LASSO) regression and generalized linear mixed models (GLMM) to adjust for institutional clustering, alongside phenotype‐treatment interaction analyses.

**Results:**

Poor outcomes occurred in 12.9% of patients. GLMM analysis demonstrated negligible institutional heterogeneity. Prognostic architectures diverged by serostatus: Ab+ outcomes were dictated by failure to achieve early functional remission (discharge mRS: OR = 3.74; CASE: OR = 1.56; both *p* < 0.01) and age ≥ 65 years (OR = 5.78, *p* = 0.040). Conversely, Ab− outcomes were independent of discharge severity metrics, driven by baseline demographic and phenotypic features: age ≥ 65 and atypical presentation. Crucially, clinical phenotype modified the therapeutic response (*p*_interaction_ = 0.025). Delayed immunotherapy (≥ 30 days) significantly increased poor outcome risk in atypical presentations (OR = 9.16, *p* = 0.025) but not in canonical phenotypes. A derived prognostic nomogram yielded excellent discrimination (AUC = 0.909).

**Discussion:**

Long‐term AE outcomes are dictated by patient‐level clinical severity and phenotype, with negligible influence from institutional variations. These findings advocate for a serostatus‐ and phenotype‐stratified management strategy. Crucially, atypical presentations exhibit a highly time‐dependent therapeutic window requiring rapid immunotherapy, while elderly patients warrant prioritized intervention to mitigate inherent baseline risks.

## 1 Introduction

Autoimmune encephalitis (AE) has emerged as a critical, treatable cause of rapidly progressive encephalopathy [1]. Despite pivotal advances in antibody discovery and immunotherapeutic protocols, the clinical trajectory of AE remains highly heterogeneous, spanning from complete recovery to permanent disability or mortality [2]. Although early immunotherapy is the cornerstone of favorable outcomes, a substantial proportion of patients remains refractory to first‐line treatments, resulting in severe long‐term neurologic sequelae [3, 4, 5]. Consequently, robust early risk stratification is imperative to guide therapeutic escalation and optimize healthcare resource allocation.

Established clinical instruments, such as the anti‐N‐methyl‐D‐aspartate receptor Encephalitis One‐Year Functional Status (NEOS) score and the Clinical Assessment Scale in Autoimmune Encephalitis (CASE), provide valuable frameworks for quantifying disease severity and predicting functional outcomes [6, 7, 8, 9, 10]. However, these tools were predominantly validated in antibody‐positive cohorts and may fail to adequately capture the prognostic profiles of the increasingly recognized “antibody‐negative” subgroup. Lacking definitive biomarker confirmation, antibody‐negative AE remains a clinical diagnosis of exclusion, representing a highly heterogeneous and diagnostically challenging syndrome [11]. Emerging evidence indicates that these patients frequently encounter diagnostic delays and inferior clinical outcomes compared to their seropositive counterparts [12, 13]. However, the specific prognostic determinants dictating their long‐term functional outcomes remain poorly characterized. Furthermore, while delayed treatment is generally considered detrimental, it remains largely unknown whether specific clinical phenotypes (canonical vs. atypical presentations) differentially modulate patient vulnerability to delayed immunotherapy.

Additionally, prior multicenter studies have largely analyzed patient cohorts as independent observations, neglecting the potential influence of “center effects”—the institutional variability in diagnostic expertise, critical care resources, and adherence to management guidelines. In complex rare diseases like AE, where treatment decisions often rely on expert consensus, institutional expertise may act as a significant modifier of patient outcomes [14, 15, 16]. Consequently, standard regression models that fail to account for this institutional clustering risk yielding biased effect estimates for individual‐level prognostic factors.

To address these critical knowledge gaps, we leveraged a large multicenter cohort of 498 patients from 11 tertiary centers in China. We employed a rigorous mixed‐effects modeling framework to account for institutional clustering and delineate the prognostic determinants of AE. Uniquely, this study sought to: (1) delineate the distinct prognostic factors of antibody‐positive versus antibody‐negative subgroups; (2) investigate whether the impact of treatment timing is modulated by clinical phenotype; and (3) quantify the contribution of institutional heterogeneity to patient outcomes. By integrating these multidimensional factors into a validated nomogram, we aim to provide a precision‐medicine tool for individualized prognostic counseling and therapeutic decision‐making.

## 2 Methods

### 2.1 Study Design and Patient Cohort

This retrospective multicenter cohort study was conducted across 11 tertiary medical centers in China, coordinated by the Affiliated Hospital of Xuzhou Medical University in collaboration with Anhui Provincial Hospital, Nanjing Brain Hospital, and eight additional participating institutions. We retrospectively reviewed the medical records of patients admitted between January 2016 and October 2024. To ensure diagnostic accuracy, all cases of autoimmune encephalitis were independently verified by at least two neurologists in strict accordance with the established clinical criteria [11]. Antibody‐positive AE was defined by the detection of neuronal surface or synaptic autoantibodies (including NMDAR, LGI1, GABABR, CASPR2, mGluR5, DPPX, AMPAR1, IgLON5, and GlyR) in cerebrospinal fluid (CSF) or serum via standardized cell‐ and tissue‐based assays. Conversely, antibody‐negative AE was defined by the absence of these recognized autoantibodies in patients who otherwise fulfilled the established clinical criteria for probable AE. To ensure cohort homogeneity, we excluded patients with alternative etiologies (infectious, toxic, metabolic, or genetic), concurrent central nervous system demyelinating disorders (AQP4‐, MOG‐, or GFAP‐IgG positivity), intracellular/onconeural antibody positivity, systemic autoimmune diseases, preexisting confounding neurological disabilities, missing critical baseline data, or loss to the one‐year follow‐up. The protocol was approved by the Ethics Committee of the Affiliated Hospital of Xuzhou Medical University (XYFY2024‐KL623‐02). Written informed consent was obtained from all participants or their legal representatives, and all procedures were conducted in strict accordance with the Declaration of Helsinki.

### 2.2 Clinical Assessment and Definitions

Baseline data were systematically extracted, encompassing demographics, clinical phenotypes, bedside neurological examination findings, and ancillary neurodiagnostics, specifically brain magnetic resonance imaging (MRI) and video‐electroencephalography (V‐EEG). The level of consciousness at presentation was quantified using the Glasgow Coma Scale (GCS). Furthermore, disease severity and functional disability were longitudinally evaluated at admission, discharge, and follow‐up utilizing the Clinical Assessment Scale in Autoimmune Encephalitis (CASE) and the modified Rankin Scale (mRS).

The primary outcome was an unfavorable functional status at 12 months, defined as an mRS score > 2 [17]. Initial clinical presentations were categorized as canonical or atypical. Canonical presentations were defined as predominant seizures, cognitive impairment, or neuropsychiatric symptoms. Atypical presentations were defined as predominant impairment of consciousness, language deficits, movement disorders, or ataxia. Regarding EEG findings, diffuse slow‐wave activity was strictly defined as sustained, generalized cortical oscillations (0.5–4 Hz) [18]. Immunotherapy was classified into first‐line (intravenous methylprednisolone, IVIG, or plasma exchange), second‐line (rituximab or cyclophosphamide), and long‐term maintenance (mycophenolate mofetil or azathioprine administered for ≥ 12 months). Delayed immunotherapy was defined as initiation ≥ 30 days after symptom onset [19]. To minimize detection bias, outcome assessors were blinded to the initial clinical severity and serostatus.

### 2.3 Statistical Analysis

Statistical analyses were performed using SPSS version 27.0 (IBM Corp., Armonk, NY) and R software (version 4.4.1). Continuous variables, expressed as means ± standard deviations or medians [P25, P75], were compared using the Mann–Whitney *U* test or Student's *t*‐test, depending on the normality of data distribution. Categorical variables, presented as frequencies (%), were compared using the chi‐square or Fisher's exact test. Missing data (restricted to 4% for CSF parameters) were imputed utilizing the Random Forest algorithm (missForest package).

A two‐stage modeling framework was employed to identify independent predictors of unfavorable outcomes. First, a Least Absolute Shrinkage and Selection Operator (LASSO) regression with 10‐fold cross‐validation was applied to select optimal predictors (*λ*_min_). Second, the retained variables were entered into multivariable fixed‐effects logistic regression and generalized linear mixed‐effects models (GLMM). In the GLMM, the treating “Hospital” was modeled as a random intercept to account for unobserved institutional clustering. Center‐level variance was quantified using the intraclass correlation coefficient (ICC), and model fit was evaluated via marginal and conditional *R* ^2^.

Furthermore, we prespecified an interaction analysis to assess whether clinical phenotypes modified the effect of delayed immunotherapy on prognosis. Stratified multivariable analyses were subsequently performed within the antibody‐positive and antibody‐negative subcohorts. Finally, a prognostic nomogram was developed based on the identified independent predictors. Nomogram performance was evaluated using the area under the curve (AUC) for discrimination and calibration plots with mean absolute error (MAE) for calibration (validated via 1000 bootstrap resamples). Two‐sided *p* values < 0.05 were considered statistically significant.

## 3 Results

### 3.1 Baseline Characteristics and Clinical Outcomes Stratified by Serostatus

The multicenter cohort included 498 patients with autoimmune encephalitis (AE), of whom 382 (76.7%) were antibody‐positive and 116 (23.3%) were antibody‐negative. Among antibody‐positive patients, the most frequent antibodies were NMDAR (*n* = 156), LGI1 (*n* = 132), GABABR (*n* = 46), CASPR2 (*n* = 20), and mGluR5 (*n* = 11). At 1‐year follow‐up, 64 patients (12.9%) had an unfavorable functional outcome (mRS score > 2), including 46 of 382 (12.0%) antibody‐positive patients (21 deaths) and 18 of 116 (15.5%) antibody‐negative patients (10 deaths).

Compared with patients with favorable outcomes (mRS ≤ 2), those with unfavorable outcomes had an older age at onset (*p* = 0.001, Table 1), lower initial GCS scores (*p* < 0.001), and higher baseline CASE and mRS scores (both *p* < 0.001). At discharge, mRS and CASE scores remained significantly higher in the unfavorable outcome group (both *p* < 0.001). Patients with unfavorable outcomes also had higher rates of intensive care unit (ICU) admission (37.5% vs. 13.8%, *p* < 0.001), comorbid tumors (23.4% vs. 6.5%, *p* < 0.001), status epilepticus (26.6% vs. 15.9%, *p* = 0.035), and use of multiple antiseizure medications (51.6% vs. 33.4%, *p* = 0.005). The proportion of patients with unfavorable outcomes was higher among those with age at onset ≥ 65 years than among those aged 19–64 years at onset (24.1% vs. 9.1%, adjusted *p* < 0.001). Similarly, patients with atypical initial presentations had a higher proportion of unfavorable outcomes than those presenting with seizures (24.6% vs. 8.7%, adjusted *p* = 0.006).

**TABLE 1:** Baseline clinical characteristics and comparative analysis stratified by one‐year functional outcome.

Characteristics	Good outcome (*n* = 434)	Poor outcome (*n* = 64)	*p*
Female, *n* (%)	183 (42.2)	25 (39.1)	0.638
Age at onset, *M* (P25, P75)	51.0 [27.0, 63.0]	60.0 [35.0, 71.0]	0.001
Age at onset			< 0.001
Age at onset, ≤ 18 years (%)	55 (12.7)	7 (10.9)	
Age at onset, 19–64 years (%)	291 (67.1)	29 (45.3)	
Age at onset, ≥ 65 years (%)	88 (20.3)	28 (43.8)	< 0.001^a^
Initial presentation, *n* (%)			0.014
Neuropsychiatric	110 (25.4)	19 (29.7)	
Seizures	189 (43.6)	18 (28.1)	
Memory dysfunction	92 (21.2)	13 (20.3)	
Atypical	43 (9.9)	14 (21.9)	0.006^b^
Delayed immunotherapy, *n* (%)	141 (32.5)	19 (29.7)	0.654
≥ 2ASM, *n* (%)	145 (33.4)	33 (51.6)	0.005
ICU admission, *n* (%)	60 (13.8)	24 (37.5)	< 0.001
GCS scores ≤ 13, *n* (%)	112 (25.8)	38 (59.4)	< 0.001
Any cognitive concerns, *n* (%)	376 (86.6)	58 (90.6)	0.373
Presence of tumor, *n* (%)	28 (6.5)	15 (23.4)	< 0.001
Diffusion restriction, *n* (%)			0.652
Cortex	117 (27.0)	23 (35.9)	
Subcortex/white matter	80 (18.4)	14 (21.9)	
Medial temporal cortex	145 (33.4)	19 (29.7)	
Infra‐tentorium	23 (5.3)	3 (4.7)	
Video‐EEG, *n* (%)			
Diffuse slow wave	98 (22.6)	20 (31.3)	0.128
Interictal epileptiform discharge	214 (49.3)	36 (56.3)	0.300
Seizures captured	61 (14.1)	7 (10.9)	0.498
Initially treated with combined first‐line IT, *n* (%)	252 (58.1)	41 (64.1)	0.363
Initially treated with second‐line IT, *n* (%)	24 (5.5)	7 (10.9)	0.100
Initially treated with long‐term IT, *n* (%)	65 (15.0)	9 (14.1)	0.848
Treated with long‐term IT during course, *n* (%)	72 (16.6)	7 (10.9)	0.248
Treated with second‐line IT during course, *n* (%)	21 (4.8)	3 (4.7)	1.000
Highest seizure frequency, *n* (%)			0.883
No	118 (27.2)	15 (23.4)	
Single	25 (5.8)	2 (3.1)	
Daily	143 (33.0)	24 (37.5)	
Weekly	103 (23.7)	16 (25.0)	
Monthly	45 (10.4)	7 (10.9)	
Type of seizure at onset, *n* (%)			
FBDS	36 (8.3)	3 (4.7)	0.316
Focal seizures	312 (71.9)	44 (68.8)	0.604
SE	69 (15.9)	17 (26.6)	0.035
Antibody positive	336 (77.4)	46 (71.9)	0.327
Hospitals, *n* (%)			0.111
Affiliated Hospital of Xuzhou Medical University	122 (28.1)	16 (25.0)	
Anhui Provincial Hospital	104 (24.0)	24 (37.5)	
Nanjing Brain Hospital	87 (20.0)	8 (12.5)	
Others	121 (27.9)	16 (25.0)	
CSF leucocyte level (10^6^/L), *M* (P25, P75)	6.0 [2.0, 16.5]	10.0 [2.0, 42.5]	0.077
CSF protein level (g/L), *M* (P25, P75)	0.39 [0.28, 0.56]	0.48 [0.30, 0.65]	0.049
Baseline CASE scores, *M* (P25, P75)	5.0 [3.0, 7.0]	7.0 [5.0, 10.0]	< 0.001
Baseline mRS scores, *M* (P25, P75)	3.0 [2.0, 3.0]	4.0 [3.0, 4.0]	< 0.001
Discharge CASE scores, *M* (P25, P75)	2.0 [1.0, 2.0]	5.0 [3.0, 10.0]	< 0.001
Discharge mRS scores, *M* (P25, P75)	2.0 [1.0, 2.0]	4.0 [3.0, 4.0]	< 0.001
GCS scores, *M* (P25, P75)	15.0 [13.0, 15.0]	13.0 [12.0, 15.0]	< 0.001
ASM number, *M* (P25, P75)	1.0 [0.0, 2.0]	2.0 [0.0, 2.0]	0.016

*Note:* Data are presented as median [P25, P75] for non‐normally distributed continuous variables and as *n* (%) for categorical variables. Non‐normally distributed continuous variables were compared using the Mann–Whitney *U* test. Categorical variables were initially compared using the Pearson *χ* ^2^ test or Fisher exact test, as appropriate. For categorical variables with more than two categories, an overall comparison was performed first. When the overall test was statistically significant, post hoc pairwise comparisons were conducted, with *p* values adjusted using the Bonferroni method. Superscript letters indicate statistically significant Bonferroni‐adjusted pairwise comparisons: ^a^age at onset ≥ 65 years versus 19–64 years; ^b^initial presentation with atypical symptoms versus seizures.

Abbreviations: ASM, antiseizure medication; CASE, Clinical Assessment Scale in Autoimmune Encephalitis; CSF, cerebrospinal fluid; EEG, electroencephalography; FBDS, faciobrachial dystonic seizures; GCS, Glasgow Coma Scale; ICU, intensive care unit; IT, immunotherapy; mRS, modified Rankin Scale; SE, status epilepticus.

In the antibody‐positive subcohort (*n* = 382), 46 patients (12.0%) had an unfavorable functional outcome at 12 months, including 19 of 156 with NMDAR antibodies (12.2%), 9 of 132 with LGI1 antibodies (6.8%), 10 of 46 with GABABR antibodies (21.7%), 1 of 20 with CASPR2 antibodies (5.0%), 5 of 11 with mGluR5 antibodies (45.5%), and 2 of 5 with DPPX antibodies (40.0%). Compared with patients with favorable outcomes, those with unfavorable outcomes had an older age at onset (*p* = 0.027, Table 2), and unfavorable outcomes were more frequent among patients with onset at age ≥ 65 years than among those with onset at age 19–64 years (19.8% vs. 8.8%, adjusted *p* = 0.015). They also had a higher proportion of admission GCS score ≤ 13 (54.4% vs. 24.7%, *p* < 0.001) and ICU admission (37.0% vs. 12.8%, *p* < 0.001), as well as higher baseline mRS scores (*p* < 0.001) and CASE scores (*p* < 0.001). At discharge, mRS and CASE scores remained significantly higher in the unfavorable outcome group (both *p* < 0.001). Tumor comorbidity was present in 28.3% (13/46) of patients with unfavorable outcomes versus 6.5% (22/336) of those with favorable outcomes (*p* < 0.001), including eight cases of lung cancer (two small‐cell lung cancers), three teratomas, one diffuse large B cell lymphoma, and one esophageal cancer. Patients with unfavorable outcomes more frequently received multiple antiseizure medications (50.0% vs. 34.2%, *p* = 0.037). Combined first‐line, second‐line, and long‐term immunotherapy was used in both groups, with no significant difference between patients with unfavorable and favorable outcomes (*p* > 0.05).

**TABLE 2:** Comparative analysis of clinical characteristics antibody positive and functional outcome.

Characteristics	Poor outcome (*n* = 46)	Good outcome (*n* = 336)	*p*
Female, *n* (%)	18 (39.1)	148 (44.1)	0.528
Age at onset, *M* (P25, P75)	60.0 [31.3, 71.3]	52.0 [28.0, 63.8]	0.027
Age at onset			0.019
Age at onset, ≤ 18 years (%)	6 (13.0)	40 (11.9)	
Age at onset, 19–64 years (%)	21 (45.7)	219 (65.2)	
Age at onset, ≥ 65 years (%)	19 (41.3)	77 (22.9)	0.015^a^
Initial presentation, *n* (%)			0.308
Neuropsychiatric	15 (32.6)	80 (23.8)	
Seizures	15 (32.6)	152 (45.2)	
Memory dysfunction	9 (19.6)	69 (20.5)	
Atypical	7 (15.2)	35 (10.4)	
Delayed immunotherapy, *n* (%)	14 (30.4)	110 (32.7)	0.754
≥ 2ASM, *n* (%)	23 (50.0)	115 (34.2)	0.037
ICU admission, *n* (%)	17 (37.0)	43 (12.8)	< 0.001
GCS scores ≤ 13, *n* (%)	25 (54.4)	83 (24.7)	< 0.001
Any cognitive concerns, *n* (%)	41 (89.1)	293 (87.2)	0.711
Presence of tumor, *n* (%)	13 (28.3)	22 (6.5)	< 0.001
Diffusion restriction, *n* (%)			0.195
Cortex	12 (26.1)	66 (19.6)	
Subcortex/white matter	7 (15.2)	39 (11.6)	
Medial temporal cortex	10 (21.7)	106 (31.6)	
Infra‐tentorium	0 (0.0)	15 (4.5)	
Video‐EEG, *n* (%)			
Diffuse slow wave	14 (30.4)	73 (21.7)	0.187
Interictal epileptiform discharge	24 (52.2)	170 (50.6)	0.841
Seizures captured	5 (10.9)	48 (14.3)	0.530
Initially treated with combined first‐line IT, *n* (%)	33 (71.7)	209 (62.2)	0.208
Initially treated with second‐line IT, *n* (%)	7 (15.2)	24 (7.1)	0.079
Initially treated with long‐term IT, *n* (%)	9 (19.6)	61 (18.2)	0.817
Treated with long‐term IT during course, *n* (%)	7 (12.5)	67 (19.9)	0.447
Treated with second‐line IT during course, *n* (%)	3 (6.5)	19 (5.7)	0.738
Highest seizure frequency, *n* (%)			0.648
No	9 (19.6)	83 (24.7)	
Single	2 (4.4)	20 (6.0)	
Daily	19 (41.3)	118 (35.1)	
Weekly	9 (19.6)	82 (24.4)	
Monthly	7 (15.2)	33 (9.8)	
Type of seizure at onset, *n* (%)			
FBDS	3 (6.5)	36 (10.7)	0.602
Focal seizures	32 (69.6)	249 (74.1)	0.512
SE	11 (23.9)	50 (14.9)	0.117
Hospitals, *n* (%)			0.204
Affiliated Hospital of Xuzhou Medical University	11 (23.9)	87 (25.9)	
Anhui Provincial Hospital	14 (30.4)	72 (21.4)	
Nanjing Brain Hospital	5 (10.9)	77 (22.9)	
Others	16 (34.8)	100 (29.8)	
CSF leucocyte level (10^6^/L), *M* (P25, P75)	13.0 [3.0, 53.3]	9.0 [2.0, 20.3]	0.053
CSF protein level (g/L), *M* (P25, P75)	0.40 [0.28, 0.60]	0.38 [0.27, 0.55]	0.299
Baseline CASE scores, *M* (P25, P75)	7.0 [5.0, 9.3]	5.0 [3.0, 7.0]	< 0.001
Baseline mRS scores, *M* (P25, P75)	4.0 [3.0, 4.0]	3.0 [2.0, 3.0]	< 0.001
Discharge CASE scores, *M* (P25, P75)	5.0 [3.0, 11.0]	2.0 [1.0, 2.0]	< 0.001
Discharge mRS scores, *M* (P25, P75)	4.0 [3.0, 4.0]	2.0 [1.0, 2.0]	< 0.001
GCS scores, *M* (P25, P75)	13.0 [12.0, 15.0]	15.0 [14.0, 15.0]	< 0.001
ASM number, *M* (P25, P75)	1.5 [0.8, 2.0]	1.0 [0.0, 2.0]	0.079

*Note:* Data are presented as median [P25, P75] for non‐normally distributed continuous variables and as *n* (%) for categorical variables. Non‐normally distributed continuous variables were compared using the Mann–Whitney *U* test. Categorical variables were initially compared using the Pearson *χ* ^2^ test or Fisher exact test, as appropriate. For categorical variables with more than two categories, an overall comparison was performed first. When the overall test was statistically significant, post hoc pairwise comparisons were conducted, with *p* values adjusted using the Bonferroni method. Superscript letters indicate statistically significant Bonferroni‐adjusted pairwise comparisons: ^a^age at onset ≥ 65 years versus 19–64 years.

Abbreviations: ASM, antiseizure medication; CASE, Clinical Assessment Scale in Autoimmune Encephalitis; CSF, cerebrospinal fluid; EEG, electroencephalography; FBDS, faciobrachial dystonic seizures; GCS, Glasgow Coma Scale; ICU, intensive care unit; IT, immunotherapy; mRS, modified Rankin Scale; SE, status epilepticus.

In the antibody‐negative subcohort (*n* = 116), 18 patients (15.5%) had an unfavorable functional outcome at 1 year. Compared with those with favorable outcomes, patients with unfavorable outcomes were older at disease onset (*p* = 0.005, Table 3). The prevalence of unfavorable outcomes was higher among patients with onset at age ≥ 65 years than among those with onset at age 19–64 years (45.0% vs. 10.0%, adjusted *p* = 0.003). Patients with atypical initial presentations also had a higher prevalence of unfavorable outcomes than those presenting with seizures (46.7% vs. 7.5%, adjusted *p* = 0.012). They were more likely to have an admission GCS score ≤ 13 (72.2% vs. 29.6%, *p* = 0.001), higher baseline mRS and CASE scores (both *p* < 0.01), and to receive multiple antiseizure medications (55.6% vs. 30.6%, *p* = 0.041). At discharge, both mRS and CASE scores remained higher in the unfavorable outcome group (both *p* < 0.001).

**TABLE 3:** Comparative analysis of clinical characteristics antibody negative and functional outcome.

Characteristics	Poor outcome (*n* = 18)	Good outcome (*n* = 98)	*p*
Female, *n* (%)	7 (38.9)	35 (35.7)	0.797
Age at onset, *M* (P25, P75)	62.5 [44.5, 71.3]	48.0 [24.8, 60.0]	0.005
Age at onset			0.001
Age at onset, ≤ 18 years (%)	1 (5.6)	15 (15.3)	
Age at onset, 19–64 years (%)	8 (44.4)	72 (73.5)	
Age at onset, ≥ 65 years (%)	9 (50.0)	11 (11.2)	0.003^a^
Initial presentation, *n* (%)			0.009
Neuropsychiatric	4 (22.2)	30 (30.6)	
Seizures	3 (16.7)	37 (37.8)	
Memory dysfunction	4 (22.2)	23 (23.5)	
Atypical	7 (38.9)	8 (8.2)	0.012^b^
Delayed immunotherapy, *n* (%)	5 (27.8)	31 (31.6)	0.747
≥ 2ASM, *n* (%)	10 (55.6)	30 (30.6)	0.041
ICU admission, *n* (%)	7 (38.9)	17 (17.4)	0.055
GCS scores ≤ 13, *n* (%)	13 (72.2)	29 (29.6)	0.001
Any cognitive concerns, *n* (%)	17 (94.4)	83 (84.7)	0.460
Presence of tumor, *n* (%)	2 (11.1)	6 (6.1)	0.608
Diffusion restriction, *n* (%)			0.763
Cortex	11 (61.1)	51 (52.0)	
Subcortex/white matter	7 (38.9)	41 (41.8)	
Medial temporal cortex	9 (50.0)	39 (39.8)	
Infra‐tentorium	3 (16.7)	8 (8.2)	
Video‐EEG, *n* (%)			
Diffuse slow wave	6 (33.3)	25 (25.5)	0.564
Interictal epileptiform discharge	12 (66.7)	44 (44.9)	0.089
Seizures captured	2 (11.1)	13 (13.3)	1.000
Initially treated with combined first‐line IT, *n* (%)	8 (44.4)	43 (43.9)	0.964
Initially treated with long‐term IT, *n* (%)	0 (0.0)	4 (4.1)	1.000
Treated with long‐term IT during course, *n* (%)	0 (0.0)	5 (5.1)	1.000
Treated with second‐line IT during course, *n* (%)	0 (0.0)	2 (2.0)	1.000
Highest seizure frequency, *n* (%)			0.344
No	6 (33.3)	35 (35.7)	
Single	0 (0.0)	5 (5.1)	
Daily	5 (27.8)	25 (25.5)	
Weekly	7 (38.9)	21 (21.4)	
Monthly	0 (0.0)	12 (12.2)	
Type of seizure at onset, *n* (%)			
Focal seizures	12 (66.7)	63 (64.3)	0.846
SE	6 (33.3)	19 (19.4)	0.215
Hospitals, *n* (%)			0.093
Affiliated Hospital of Xuzhou Medical University	5 (27.8)	35 (35.7)	
Anhui Provincial Hospital	10 (55.6)	32 (32.7)	
Nanjing Brain Hospital	3 (16.7)	13 (13.3)	
Others	0 (0.0)	18 (18.4)	
CSF leucocyte level (10^6^/L), *M* (P25, P75)	2.0 [2.0, 22.5]	3.0 [1.0, 8.0]	0.501
CSF protein level (g/L), *M* (P25, P75)	0.60 [0.40, 0.80]	0.41 [0.30, 0.63]	0.060
Baseline CASE scores, *M* (P25, P75)	7.5 [4.8, 10.3]	5.0 [3.0, 6.0]	0.002
Baseline mRS scores, *M* (P25, P75)	4.0 [3.0, 4.0]	3.0 [2.0, 3.0]	< 0.001
Discharge CASE scores, *M* (P25, P75)	5.0 [2.8, 10.5]	2.0 [1.0, 2.0]	< 0.001
Discharge mRS scores, *M* (P25, P75)	3.5 [3.0, 4.3]	2.0 [1.0, 2.0]	< 0.001
GCS scores, *M* (P25, P75)	12.0 [10.5, 14.0]	15.0 [13.0, 15.0]	< 0.001
ASM number, *M* (P25, P75)	2.0 [0.0, 2.3]	1.0 [0.0, 2.0]	0.067

*Note:* Data are presented as median [P25, P75] for non‐normally distributed continuous variables and as *n* (%) for categorical variables. Non‐normally distributed continuous variables were compared using the Mann–Whitney *U* test. Categorical variables were initially compared using the Pearson *χ* ^2^ test or Fisher exact test, as appropriate. For categorical variables with more than two categories, an overall comparison was performed first. When the overall test was statistically significant, post hoc pairwise comparisons were conducted, with *p* values adjusted using the Bonferroni method. Superscript letters indicate statistically significant Bonferroni‐adjusted pairwise comparisons: ^a^age at onset ≥ 65 years versus 19–64 years; ^b^initial presentation with atypical symptoms versus seizures.

Abbreviations: ASM, antiseizure medication; CASE, Clinical Assessment Scale in Autoimmune Encephalitis; CSF, cerebrospinal fluid; EEG, electroencephalography; FBDS, faciobrachial dystonic seizures; GCS, Glasgow Coma Scale; ICU, intensive care unit; IT, immunotherapy; mRS, modified Rankin Scale; SE, status epilepticus.

### 3.2 Multilevel Determinants of Prognosis in the Overall Cohort

To address multicollinearity and ensure model parsimony, a two‐stage analytical framework was applied. Least absolute shrinkage and selection operator (LASSO) regression with 10‐fold cross‐validation was utilized to screen candidate variables, selecting the penalty parameter that minimized the mean cross‐validated error (*λ*_min_, Figure 1A). The retained variables were subsequently entered in a multivariable fixed‐effects logistic regression model (*n* = 498). In this fixed‐effects model, higher discharge CASE scores (OR = 1.50, 95% CI 1.23–1.89, *p* < 0.001, Figure 1B) and mRS scores (OR = 2.81, 95% CI 1.79–4.53, *p* < 0.001) were independently associated with unfavorable outcomes. Additionally, age at onset ≥ 65 years (OR = 5.66, 95% CI 1.55–25.16, *p* = 0.014) and atypical initial presentation (OR = 3.89, 95% CI 1.27–12.10, *p* = 0.017) were also independently associated with unfavorable outcomes.

**FIGURE 1:**
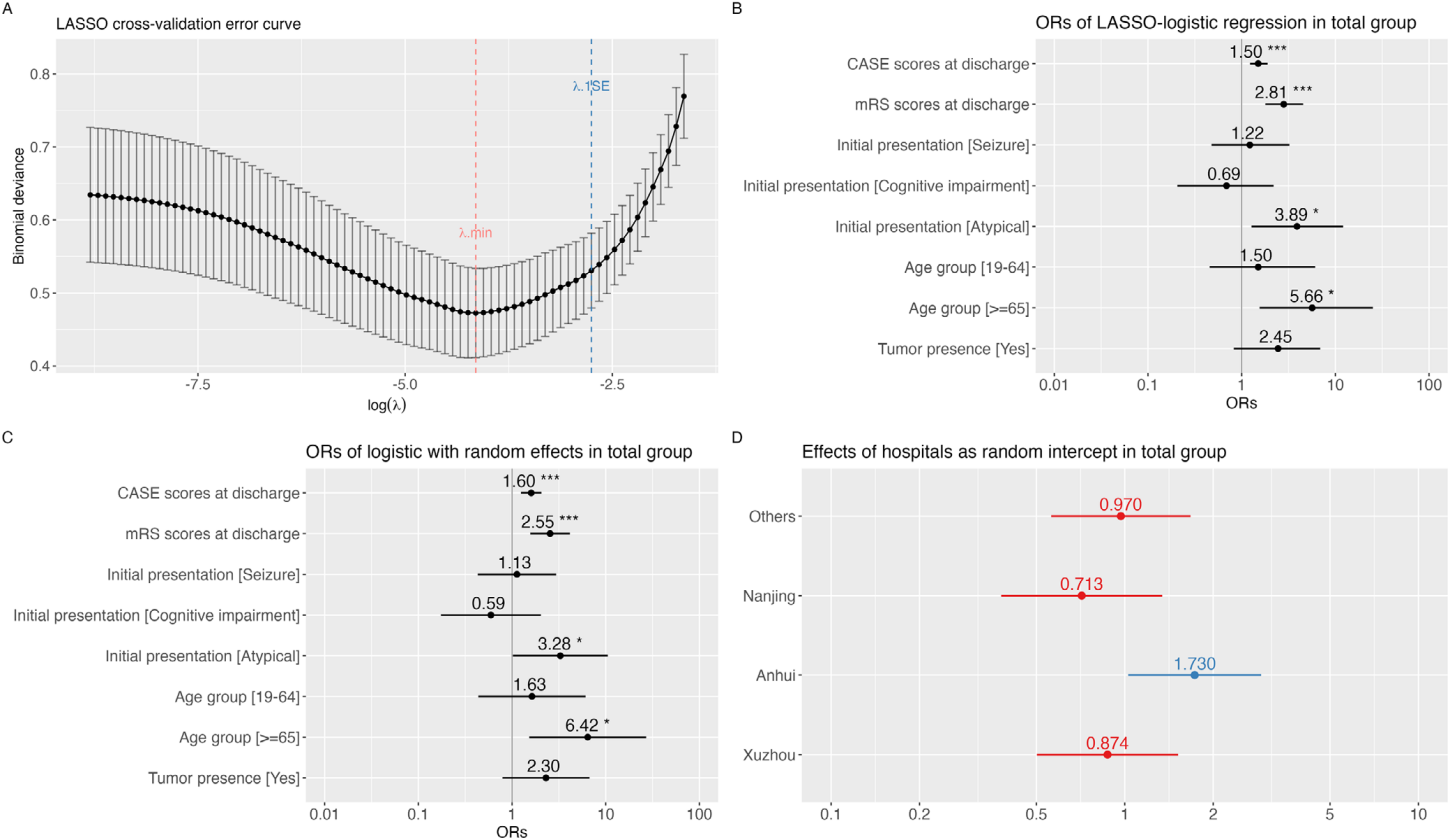
Variable selection and multivariable prognostic models in the autoimmune encephalitis cohort. (A) Least Absolute Shrinkage and Selection Operator (LASSO) feature selection for the autoimmune encephalitis cohort (*n* = 498). The *x*‐axis represents log(*λ*), and the *y*‐axis represents the binomial deviance. Error bars indicate ±1 standard error of the 10‐fold cross‐validation estimate. The red dashed line indicates the *λ* value that yields the minimum cross‐validation error. The blue dashed line indicates the largest *λ* value within one standard error of the minimum. (B) Forest plot of the fixed‐effects logistic regression model. Data are presented as odds ratios (ORs) and 95% confidence intervals (CIs) for predictors of unfavorable outcome. (C) Forest plot of the generalized linear mixed‐effects model (GLMM) incorporating hospital center as a random intercept, displaying adjusted ORs and 95% CIs for predictors of unfavorable outcome. (D) Plot of hospital‐specific random effects from the GLMM, presented as ORs and 95% CIs. Xuzhou, the Affiliated Hospital of Xuzhou Medical University; Anhui, Anhui Provincial Hospital; Nanjing, Nanjing Brain Hospital; other institutions, eight additional participating institutions. CASE, Clinical Assessment Scale in Autoimmune Encephalitis; CI, confidence interval; GLMM, generalized linear mixed‐effects model; LASSO, Least Absolute Shrinkage and Selection Operator; mRS, modified Rankin Scale; OR, odds ratio. Significance levels: *p < 0.05, ***p < 0.001.

To account for potential institutional clustering, we constructed a mixed‐effects logistic regression model with the hospital as a random intercept. In this model, discharge CASE score (OR = 1.60, 95% CI 1.25–2.06, *p* < 0.001, Figure 1C) and mRS score (OR = 2.55, 95% CI 1.57–4.15, *p* < 0.001) remained independently associated with unfavorable outcomes. Furthermore, age at onset ≥ 65 years (OR = 6.42, 95% CI 1.53–27.02, *p* = 0.011) and atypical initial presentation (OR = 3.28, 95% CI 1.02–10.52, *p* = 0.046) were also retained as independent predictors. The intraclass correlation coefficient (ICC) for the mixed‐effects model was 0.055 (*τ*_00_ = 0.192, *p* = 0.23, Figure 1D), indicating that 5.5% of outcome variance was attributable to between‐center differences. Model explanatory performance was reflected by a conditional *R* ^2^ of 0.674 and a marginal *R* ^2^ of 0.655.

### 3.3 Interaction Analysis: Clinical Phenotype and Delayed Immunotherapy

In the overall cohort (*n* = 498), multivariable logistic regression models including phenotype‐by‐treatment interaction terms were used to assess whether clinical phenotype modified the association between delayed immunotherapy and 12‐month unfavorable outcome. A statistically significant interaction was identified for atypical initial presentation (*p*_interaction_ = 0.024, Figure 2A). In patients with atypical presentations, delayed immunotherapy was associated with higher odds of unfavorable outcome (OR = 9.32, 95% CI 1.34–66.65, *p* = 0.024, Figure 2A,C). In contrast, no statistically significant interaction was observed in patients with canonical phenotypes (seizures, cognitive impairment, or neuropsychiatric symptoms). Mixed‐effects logistic regression with hospital as a random intercept further confirmed the interaction in the atypical presentation subgroup (OR = 9.16, 95% CI 1.31–63.93, *p* = 0.025, Figure 2B). The intraclass correlation coefficient was 0.043 (*τ*_00_ = 0.149, *p* = 0.35).

**FIGURE 2:**
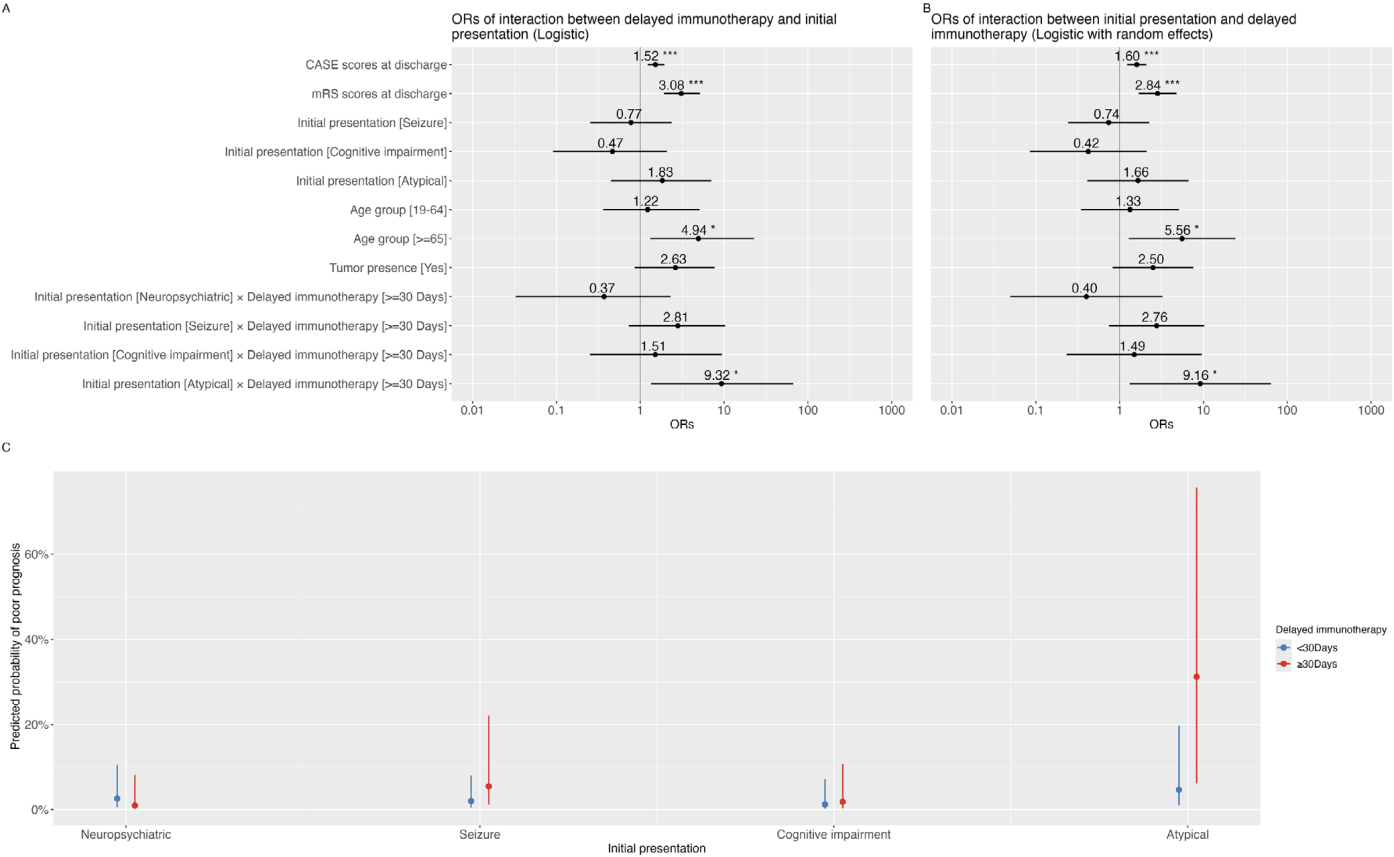
Interaction between initial clinical phenotype and delayed immunotherapy. (A) Forest plot of the multivariable fixed‐effects logistic regression model. Data are presented as adjusted odds ratios (ORs) and 95% confidence intervals (CIs) for main effects and the interaction terms between initial clinical presentation and delayed immunotherapy (≥ 30 days). (B) Forest plot of the multivariable generalized linear mixed‐effects model with hospital center as a random intercept. Data are presented as adjusted ORs and 95% CIs for the interaction analysis, accounting for center‐level clustering. (C) Predicted probability of poor prognosis stratified by initial clinical presentation and timing of immunotherapy (< 30 days [blue] vs. ≥ 30 days [red]). Data points represent estimated probabilities, and error bars indicate 95% CIs. CASE, Clinical Assessment Scale in Autoimmune Encephalitis; CI, confidence interval; mRS, modified Rankin Scale; OR, odds ratio. Significance levels: *p < 0.05, ***p < 0.001.

### 3.4 Distinct Prognostic Factors Stratified by Serostatus

To delineate prognostic determinants by serostatus, predictor variables were initially selected via LASSO regression with 10‐fold cross‐validation (*λ*_min_, Figures 3A and 4A). Retained variables were subsequently incorporated into fixed‐effects multivariable logistic regression and generalized linear mixed models within the antibody‐positive (*n* = 382) and antibody‐negative (*n* = 116) subcohorts, enabling the estimation of independent effects while accounting for center‐level variance.

**FIGURE 3:**
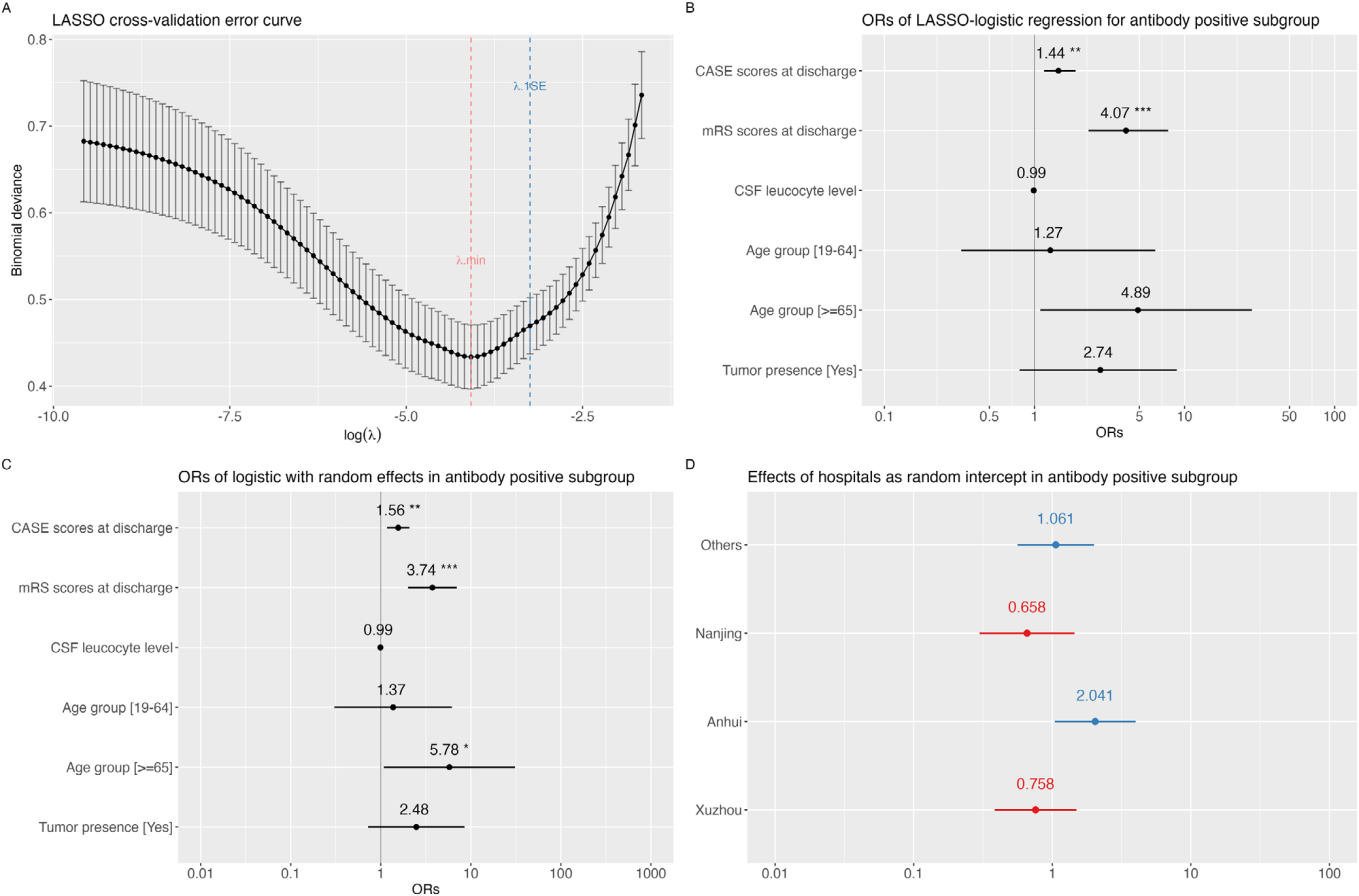
Variable selection and multivariable prognostic models in the antibody‐positive autoimmune encephalitis cohort. (A) Least Absolute Shrinkage and Selection Operator (LASSO) feature selection for the antibody‐positive cohort (*n* = 382). The *x*‐axis represents log(*λ*), and the *y*‐axis represents the binomial deviance. Error bars indicate ±1 standard error of the 10‐fold cross‐validation estimate. The red dashed line indicates the *λ* value that yields the minimum cross‐validation error. The blue dashed line indicates the largest *λ* value within one standard error of the minimum. (B) Forest plot of the fixed‐effects logistic regression model. Data are presented as odds ratios (ORs) and 95% confidence intervals (CIs) for predictors of unfavorable outcome. (C) Forest plot of the generalized linear mixed‐effects model (GLMM) with hospital center included as a random intercept, showing adjusted ORs and 95% CIs for predictors of unfavorable outcome. (D) Plot of hospital‐specific random effects from the GLMM, presented as ORs and 95% CIs. Xuzhou, the Affiliated Hospital of Xuzhou Medical University; Anhui, Anhui Provincial Hospital; Nanjing, Nanjing Brain Hospital; other institutions, eight additional participating institutions. CASE, Clinical Assessment Scale in Autoimmune Encephalitis; CI, confidence interval; GLMM, generalized linear mixed‐effects model; LASSO, Least Absolute Shrinkage and Selection Operator; mRS, modified Rankin Scale; OR, odds ratio. Significance levels: *p < 0.05, **p < 0.01, ***p < 0.001.

**FIGURE 4:**
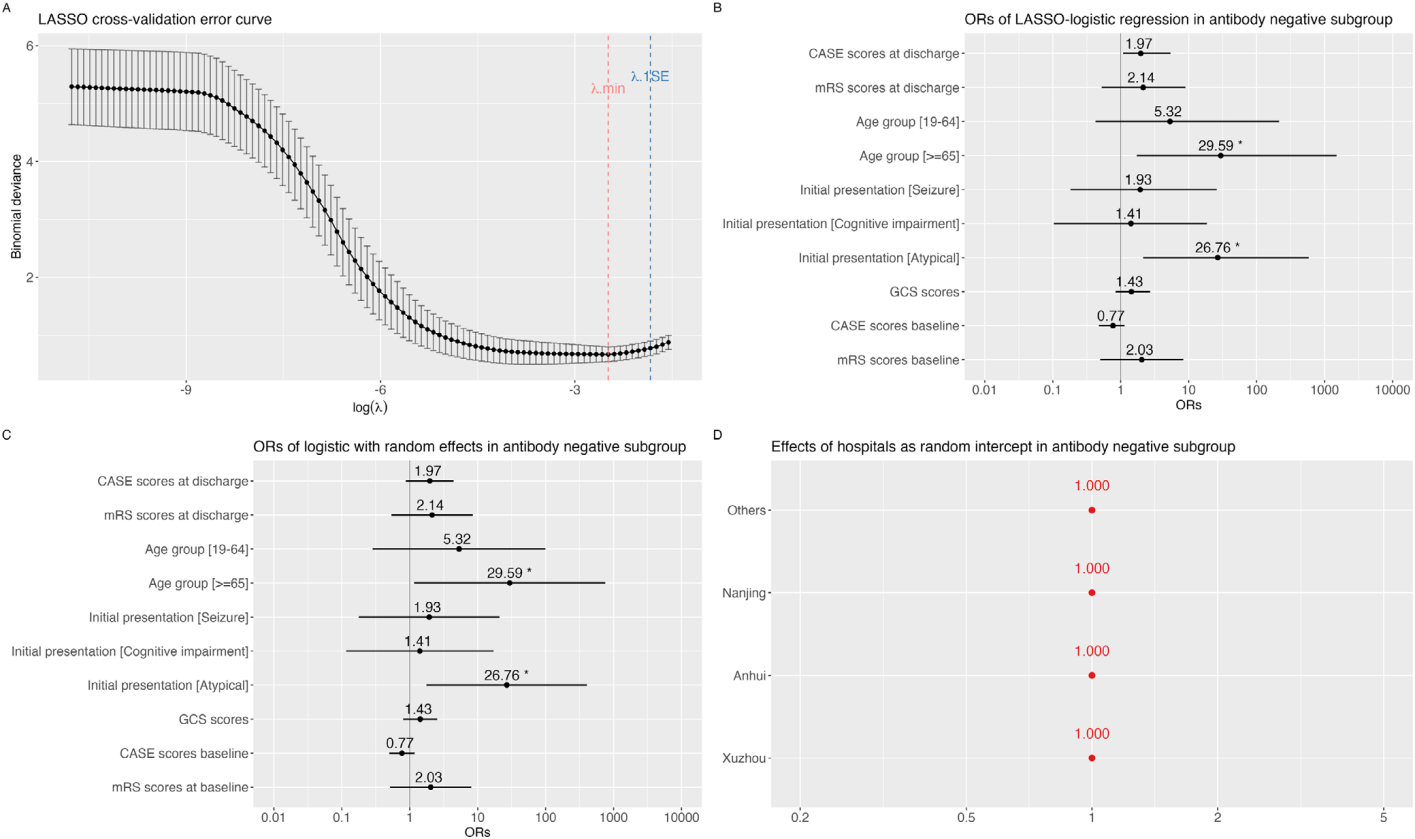
Variable selection and multivariable prognostic models in the antibody‐negative autoimmune encephalitis cohort. (A) Least Absolute Shrinkage and Selection Operator (LASSO) feature selection for the antibody‐negative cohort (*n* = 116). The *x*‐axis represents log(*λ*), and the *y*‐axis represents the binomial deviance. Error bars indicate ±1 standard error of the 10‐fold cross‐validation estimate. The red dashed line indicates the *λ* value that yields the minimum cross‐validation error. The blue dashed line indicates the largest *λ* value within one standard error of the minimum. (B) Forest plot of the fixed‐effects logistic regression model. Data are presented as odds ratios (ORs) and 95% confidence intervals (CIs) for predictors of unfavorable outcome. (C) Forest plot of the generalized linear mixed‐effects model (GLMM) with hospital center included as a random intercept, showing adjusted ORs and 95% CIs for predictors of unfavorable outcome. (D) Plot of hospital‐specific random effects from the GLMM, presented as ORs and 95% CIs. Xuzhou, the Affiliated Hospital of Xuzhou Medical University; Anhui, Anhui Provincial Hospital; Nanjing, Nanjing Brain Hospital; other institutions, eight additional participating institutions. CASE, Clinical Assessment Scale in Autoimmune Encephalitis; CI, confidence interval; GLMM, generalized linear mixed‐effects model; LASSO, Least Absolute Shrinkage and Selection Operator; mRS, modified Rankin Scale; OR, odds ratio. Significance levels: *p < 0.05.

In the antibody‐positive subgroup, the fixed‐effects model identified higher discharge CASE (OR = 1.44, 95% CI 1.16–1.88, *p* = 0.003, Figure 3B) and mRS scores (OR = 4.18, 95% CI 2.33–8.04, *p* < 0.001) as predictors of unfavorable outcomes. In the mixed‐effects model, discharge CASE (OR = 1.56, 95% CI 1.17–2.08, *p* = 0.002, Figure 3C) and mRS (OR = 3.74, 95% CI 2.01–6.98, *p* < 0.001) remained statistically significant predictors of unfavorable outcome. Age at onset ≥ 65 years was also independently associated with unfavorable outcome in the mixed‐effects model (OR = 5.78, 95% CI 1.08–30.95, *p* = 0.040). The intraclass correlation coefficient for center‐level variance was 0.088 (*τ*_00_ = 0.318, *p* = 0.22, Figure 3D).

In the antibody‐negative subgroup, discharge CASE and discharge mRS scores were not significantly associated with 12‐month unfavorable outcome in either fixed‐effects or mixed‐effects models (all *p* > 0.05). In contrast, atypical initial presentation was independently associated with unfavorable outcome in the fixed‐effects model (OR = 18.41, 95% CI 1.28–463.83, *p* = 0.045, Figure 4B) and in the mixed‐effects model (OR = 26.77, 95% CI 1.76–406.72, *p* = 0.018, Figure 4C). Age at onset ≥ 65 years was also independently associated with unfavorable outcome in the fixed‐effects model (OR = 34.71, 95% CI 1.80–2289.54, *p* = 0.042) and in the mixed‐effects model (OR = 29.59, 95% CI 1.16–755.94, *p* = 0.040). The ICC was approximately 0.000 (*p* = 1.000, Figure 4D).

### 3.5 Development and Validation of a Prognostic Nomogram

A prognostic nomogram was constructed to estimate the individual probability of 1‐year poor functional outcome (mRS > 2) (Figure 5A). The model incorporated discharge CASE score, discharge mRS score, initial clinical phenotype, age strata, and tumor comorbidity, based on multivariable logistic regression. Individual predictor values were assigned weighted points, and the total score corresponded to a predicted probability of poor outcome. Internal validation was performed using bootstrap resampling with 1000 iterations. Calibration analysis demonstrated a mean absolute error of 0.022, and the calibration curve showed concordance between predicted and observed probabilities (Figure 5B). The model demonstrated good discrimination for unfavorable outcome, with an AUC of 0.909 (95% CI 0.86–0.96, Figure 5C).

**FIGURE 5:**
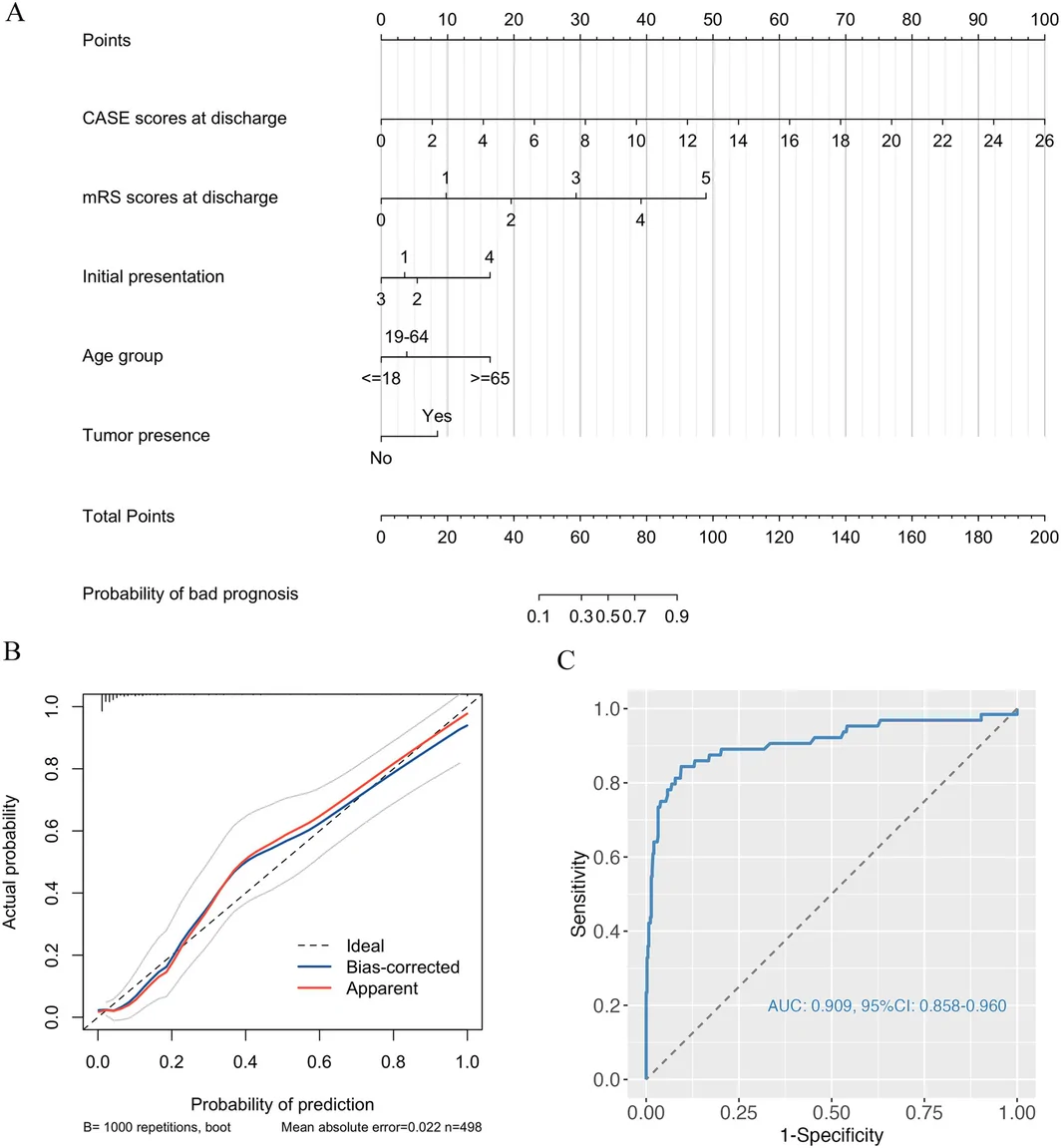
Prognostic nomogram and internal validation models. (A) Nomogram developed from a multivariable logistic regression model to predict the probability of poor prognosis. Individual patient values for discharge severity (CASE and mRS scores), initial clinical presentation (1, neuropsychiatric; 2, seizures; 3, cognitive impairment; 4, atypical presentation), age group, and tumor presence are assigned a specific point value on the top axis. The sum of these points on the “Total Points” axis corresponds to the predicted probability of poor prognosis on the bottom axis. (B) Calibration curve evaluating the agreement between predicted and actual probabilities using 1000 bootstrap resamples (*n* = 498). The dashed 45‐degree line represents ideal perfect prediction. The red solid line indicates apparent accuracy, and the blue solid line indicates bias‐corrected accuracy (mean absolute error = 0.022). (C) Receiver operating characteristic (ROC) curve for the predictive model. The area under the curve (AUC) is 0.909 (95% CI 0.86–0.96). AUC, area under the curve; CASE, Clinical Assessment Scale in Autoimmune Encephalitis; mRS, modified Rankin Scale; ROC, receiver operating characteristic.

## 4 Discussion

In this large‐scale multicenter cohort study, we utilized a mixed‐effects modeling framework to evaluate prognostic factors in AE. Our analysis yielded three primary clinical insights. First, we observed distinct prognostic profiles stratified by serostatus: while 1‐year outcomes in antibody‐positive AE were closely associated with functional severity at discharge, outcomes in antibody‐negative AE were predominantly driven by age at onset ≥ 65 years and atypical initial presentation, independent of standard discharge severity metrics. Second, we identified a phenotype‐specific interaction regarding treatment delay. Specifically, delayed immunotherapy (≥ 30 days) was associated with significantly higher odds of unfavorable outcomes exclusively in patients with atypical presentations. Third, the low intraclass correlation coefficients across models indicated that patient‐level clinical characteristics, rather than institutional heterogeneity, are the primary determinants of AE prognosis. Building on these findings, we developed a prognostic nomogram (AUC = 0.909) to facilitate personalized risk stratification.

A key finding of this study is the delineation of distinct prognostic profiles for antibody‐positive and antibody‐negative subgroups. In the antibody‐positive cohort, while initial severe presentations (admission GCS ≤ 13) and tumor comorbidity were associated with poor outcomes univariately, the persistence of clinical impairment at discharge (higher mRS and CASE scores) and older age at onset (≥ 65 years) emerged as the primary independent predictors of long‐term disability. This aligns with previous studies suggesting that failure to achieve early clinical remission is a critical determinant of poor long‐term outcomes [20, 21]. Furthermore, although the intensity of immunotherapy did not significantly differ between outcome groups, a subset of patients remained refractory. This trajectory may reflect irreversible neuronal injury—potentially exacerbated by underlying malignancies or advanced age—rather than inadequate immunologic treatment [22, 23].

In contrast, antibody‐negative AE showed a potentially distinct prognostic profile. In this subgroup, discharge CASE and mRS scores were not independently associated with 1‐year outcomes, suggesting that the prognostic utility of these measures may vary across AE subgroups. Instead, older age at onset (≥ 65 years) and atypical initial presentations were associated with unfavorable outcomes, with negligible between‐center variance. However, given the limited sample size, small number of outcome events, and imprecision of the estimated associations, these findings should be interpreted cautiously and require confirmation in larger independent cohorts. Furthermore, given that patients aged ≥ 65 years and those aged < 65 years showed no significant differences in rates of delayed immunotherapy, tumor comorbidity, or treatment regimens, these outcomes may be less driven by standard treatment‐related variables. Consequently, the clinical course in seronegative patients might be more heavily influenced by intrinsic patient‐level vulnerabilities—such as T cell–mediated cytotoxicity or concurrent age‐related neurodegenerative processes—rather than the typically reversible synaptic dysfunction characteristic of antibody‐mediated disease [5, 13, 24, 25, 26]. The limited prognostic utility of standard severity metrics in this subpopulation underscores the need to identify novel, subtype‐specific biomarkers.

Our interaction analysis revealed that the prognostic impact of delayed immunotherapy varies by clinical phenotype. Specifically, delayed treatment (≥ 30 days) was associated with an approximately ninefold increase in the odds of an unfavorable outcome in patients with atypical presentations, whereas this association was not statistically significant in those with canonical phenotypes. Clinically, canonical manifestations such as seizures typically prompt immediate medical evaluation and facilitate earlier intervention. In contrast, atypical presentations frequently result in diagnostic latency due to prolonged psychiatric or metabolic workups [15, 27]. Our data suggest that patients with atypical phenotypes may possess a narrower therapeutic window, rendering them particularly vulnerable to delayed immunologic intervention. These findings highlight the necessity of considering early or empiric immunotherapy for patients with unexplained encephalopathy or movement disorders, even in the absence of classic limbic symptoms [28].

Previous studies in Western cohorts have suggested that specialized neuroimmunology centers may yield superior AE outcomes [15]. In our cohort, however, the mixed‐effects analysis demonstrated a low and nonsignificant intraclass correlation coefficient (overall ICC = 0.055, *p* = 0.23), indicating minimal inter‐hospital variation in 1‐year prognosis. This uniformity may reflect the relatively homogenized care practices across the participating tertiary centers in China, potentially facilitated by the recent implementation of standardized AE consensus guidelines [11]. Analytically, this finding emphasizes that patient‐level clinical and demographic characteristics—rather than institutional heterogeneity—are the primary determinants of prognosis. Consequently, while referral to specialized centers remains essential for timely diagnosis and access to complex immunotherapies, strategies to improve long‐term outcomes must predominantly focus on the early recognition and targeted management of intrinsic, patient‐specific risk factors, particularly advanced age, atypical clinical phenotypes, and tumor comorbidities.

To translate these findings into clinical practice, we developed and internally validated a prognostic nomogram incorporating the identified predictors: discharge CASE and mRS scores, initial clinical phenotype, age, and tumor comorbidity. Demonstrating high discriminative accuracy (AUC = 0.909) and robust calibration, this instrument facilitates individualized risk stratification at the bedside. By identifying high‐risk patients—particularly older adults with atypical presentations or those with substantial residual deficits at discharge—the nomogram may help guide targeted surveillance and personalized rehabilitation strategies.

Several limitations of this study should be acknowledged. First, the retrospective design inherently carries the risk of selection bias and unmeasured confounding. While our mixed‐effects framework robustly accounted for unobserved center‐level clustering, it cannot entirely eliminate residual patient‐level biases. Second, the antibody‐negative cohort is inherently heterogeneous, and its relatively small sample size (*n* = 116) yielded wide confidence intervals for key predictors. Accordingly, the precise magnitudes of these associations should be interpreted cautiously. Furthermore, as novel autoantibodies continue to be identified, future reclassification of currently seronegative patients may further refine these prognostic profiles. Third, owing to sample size constraints within subgroups, we analyzed treatments using broad classifications—specifically first‐line versus second‐line or long‐term modalities—rather than evaluating the comparative efficacy of individual immunotherapeutic agents such as rituximab or cyclophosphamide. Finally, our cohort was drawn exclusively from tertiary referral centers, which may introduce referral bias and limit the generalizability of our findings to community hospital settings.

In conclusion, this multicenter study suggests that prognostic factors in AE may vary by serostatus and clinical phenotype. We provide evidence that antibody‐negative AE follows a distinct clinical trajectory predominantly associated with advanced age and atypical presentations, rather than standard discharge severity metrics. By identifying a phenotype‐specific vulnerability to delayed immunotherapy and developing a validated prognostic nomogram, our findings facilitate individualized risk stratification. These insights underscore the critical need for rapid recognition of atypical phenotypes and targeted, early interventions to optimize outcomes, particularly in high‐risk older adults.

## Author Contributions

Qingwei Lai and Sen Qun designed and supervised the study. Qingwei Lai, Xiaohong Tang, Jingxian Wang, Kaixin Zhu, Zhen Zhang, Liao Wu, and Wei Wang drafted and revised the manuscript. Chang Li, Qingyun Li, Hao Li, Jinghua Chen, Zhangxu Lian, Bin Liu, Tengfei Liu, Nuan Wang, and Chunmei Wen acquired the data. Lishun Xiao performed data analysis and interpretation. All authors contributed to the article and approved the submitted version.

## Funding

This study was supported financially by Clinical Research Special Project for High‐Level Hospital Construction in Jiangsu Province (LCZX202513), Science and Technology Program of Xuzhou (KC25064), Paired Assistance Research Project of The Affiliated Hospital of Xuzhou Medical University (SHJDBF2024206), XZHMU‐QL Joint Research Fund (QL‐YB077), and Special Funding for Overseas Research and Training for Staff of the Affiliated Hospital of Xuzhou Medical University.

## Conflicts of Interest

The authors declare no conflicts of interest.

## Data Availability

The data that support the findings of this study are available from the corresponding author upon reasonable request.

## References

[1] J. Dalmau and F. Graus , “Antibody‐Mediated Encephalitis,” New England Journal of Medicine 378, no. 9 (2018): 840–851, 10.1056/NEJMra1708712. 29490181

[2] M. J. Titulaer , L. McCracken , I. Gabilondo , et al., “Treatment and Prognostic Factors for Long‐Term Outcome in Patients With Anti‐NMDA Receptor Encephalitis: An Observational Cohort Study,” Lancet Neurology 12, no. 2 (2013): 157–165, 10.1016/S1474-4422(12)70310-1. PMC3563251 23290630

[3] S. Thakolwiboon , M. Gilligan , E. Orozco , et al., “Autoimmune Encephalitis: Recovery, Residual Symptoms and Predictors of Long‐Term Sequelae,” Journal of Neurology, Neurosurgery, and Psychiatry 96, no. 8 (2025): 736–743, 10.1136/jnnp-2024-334957. PMC12462787 39832911

[4] C. Milano , E. Saylam , C. Papi , et al., “Clinical Characterization and Long‐Term Outcome in Children and Adults With Anti‐AMPA Receptor Encephalitis,” Neurology: Neuroimmunology & Neuroinflammation 12, no. 5 (2025): e200453. 10.1212/NXI.0000000000200453 PMC12275903 40680249

[5] F. Li , Y. He , X. Chen , A. Yang , J. Zhang , and W. Zang , “Clinical Spectrum and Long‐Term Outcomes of Antibody‐Negative Severe Autoimmune Encephalitis: A Retrospective Study,” Frontiers in Immunology 16 (2025): 1591771, 10.3389/fimmu.2025.1591771. PMC12119582 40443652

[6] M. Nikolaus , P. Rausch , K. Rostásy , et al., “Retrospective Pediatric Cohort Study Validates NEOS Score and Demonstrates Applicability in Children With Anti‐NMDAR Encephalitis,” Neurology: Neuroimmunology & Neuroinflammation 10, no. 3 (2023): e200102. 10.1212/NXI.0000000000200102 PMC10032577 36948591

[7] M. Mazowiecki , L. Flet‐Berliac , J. Roux , et al., “Long‐Term Clinical and Biological Prognostic Factors of Anti‐NMDA Receptor Encephalitis in Children,” Neurology: Neuroimmunology & Neuroinflammation 12, no. 2 (2025): e200346, 10.1212/NXI.0000000000200346. PMC12231184 39715492

[8] S. Macher , G. Bsteh , R. Höftberger , T. Berger , P. Rommer , and T. Zrzavy , “Clinical Scales in Autoimmune Encephalitis—A Retrospective Monocentric Cohort Study,” Annals of Clinical Translational Neurology 10, no. 10 (2023): 1768–1775, 10.1002/acn3.51865. PMC10578879 37545101

[9] Q. Lai , Y. Chen , W. Wang , Z. Lian , T. Liu , and C. Wen , “Identifying Key Prognostic Indicators for Relapse and Chronic Epilepsy in Autoimmune Encephalitis: Insights From a Multicenter Retrospective Study,” Journal of Inflammation Research 17 (2024): 11529–11543, 10.2147/JIR.S481729. PMC11681903 39735892

[10] H. Abboud , F. Briggs , R. Buerki , et al., “Residual Symptoms and Long‐Term Outcomes After All‐Cause Autoimmune Encephalitis in Adults,” Journal of the Neurological Sciences 434 (2022): 120124, 10.1016/j.jns.2021.120124. 34998237

[11] F. Graus , M. J. Titulaer , R. Balu , et al., “A Clinical Approach to Diagnosis of Autoimmune Encephalitis,” Lancet Neurology 15, no. 4 (2016): 391–404, 10.1016/S1474-4422(15)00401-9. PMC5066574 26906964

[12] G. Mangioris , E. Orozco , D. Dubey , et al., “Long‐Term Outcomes in Antibody‐Negative Autoimmune Encephalitis: A Retrospective Study,” Journal of Neurology 271, no. 12 (2024): 7502–7515, 10.1007/s00415-024-12680-y. PMC12477645 39278895

[13] L. Di Cosmo , S. Mulic‐Al Bunni , Y. Goh , et al., “Systematic Review and Meta‐Analysis of the Clinical Features Associated With Seronegative Autoimmune Encephalitis,” Neurology: Neuroimmunology & Neuroinflammation 13, no. 2 (2026): e200540, 10.1212/NXI.0000000000200540. PMC12794537 41499723

[14] W. Lee , S. Lee , S. Hong , et al., “Immunotherapy in Probable Autoimmune Encephalitis Without Detected Autoantibody,” Journal of Immunology 204, no. 1S (2020): 160.19.

[15] E. P. Flanagan , M. D. Geschwind , A. S. Lopez‐Chiriboga , et al., “Autoimmune Encephalitis Misdiagnosis in Adults,” JAMA Neurology 80, no. 1 (2023): 30–39, 10.1001/jamaneurol.2022.4251. PMC9706400 36441519

[16] J. Dalmau and F. Graus , “Diagnostic Criteria for Autoimmune Encephalitis: Utility and Pitfalls for Antibody‐Negative Disease,” Lancet Neurology 22, no. 6 (2023): 529–540, 10.1016/S1474-4422(23)00083-2. 37210100

[17] J. Brenner , A. E. M. Bastiaansen , M. Guasp , et al., “Development and Validation of the NEOS2 Score for Prediction of Long‐Term Outcomes and Improvement After First‐Line Immunotherapy in Patients With Anti‐NMDAR Encephalitis: An International Cohort Study,” Lancet Regional Health–Europe 62 (2026): 101562, 10.1016/j.lanepe.2025.101562. PMC12757447 41488792

[18] M. Yildirim , B. Konuskan , D. Yalnizoglu , H. Topaloglu , I. Erol , and B. Anlar , “Electroencephalographic Findings in Anti‐*N*‐Methyl‐D‐Aspartate Receptor Encephalitis in Children: A Series of 12 Patients,” Epilepsy & Behavior 78 (2018): 118–123, 10.1016/j.yebeh.2017.09.022. 29179102

[19] X. Liu , K. Guo , J. Lin , et al., “Long‐Term Seizure Outcomes in Patients With Autoimmune Encephalitis: A Prospective Observational Registry Study Update,” Epilepsia 63, no. 7 (2022): 1812–1821, 10.1111/epi.17245. 35357695

[20] X. Zhao , N. Wang , T. Yang , et al., “Systemic and Organ‐Specific Autoantibodies and Worse Outcomes in Autoimmune Encephalitis: A Multicenter Cohort Study,” Frontiers in Immunology 17 (2026): 1759213, 10.3389/fimmu.2026.1759213. PMC12867786 41646854

[21] R. Wesselingh , S. Griffith , K. Ko , et al., “Biomarkers of Neuroaxonal and Astrocytic Pathology in Autoimmune Encephalitis,” Brain, Behavior, and Immunity 132 (2026): 106206, 10.1016/j.bbi.2025.106206. 41354225

[22] W. Song , K. Li , J. Li , et al., “Thymoma‐Associated Autoimmune Encephalitis: Analysis of Factors Determining Prognosis,” CNS Neuroscience & Therapeutics 29, no. 5 (2023): 1213–1221, 10.1111/cns.14166. PMC10068466 36914970

[23] F. Lamblin , J. Kerstens , S. Muniz‐Castrillo , et al., “Comparative Study of Paraneoplastic and Nonparaneoplastic Autoimmune Encephalitis With GABA_B_R Antibodies,” Neurology: Neuroimmunology & Neuroinflammation 11, no. 3 (2024): e200229, 10.1212/NXI.0000000000200229. PMC11087025 38657198

[24] F. Mannara , M. Radosevic , J. Planagumà , et al., “Allosteric Modulation of NMDA Receptors Prevents the Antibody Effects of Patients With Anti‐NMDAR Encephalitis,” Brain 143, no. 9 (2020): 2709–2720, 10.1093/brain/awaa195. 32830245

[25] T. Kirschstein and R. Köhling , “Functional Changes in Neuronal Circuits due to Antibody‐Driven Autoimmune Response,” Neurobiology of Disease 184 (2023): 106221, 10.1016/j.nbd.2023.106221. 37414365

[26] D. Frieser , A. Pignata , L. Khajavi , et al., “Tissue‐Resident CD8(+) T Cells Drive Compartmentalized and Chronic Autoimmune Damage Against CNS Neurons,” Science Translational Medicine 14, no. 640 (2022): eabl6157, 10.1126/scitranslmed.abl6157. 35417189

[27] D. Martins , R. Faria , S. Rodrigues , and M. Pinho , “Psychiatric Manifestations of Anti‐NMDAR Encephalitis,” European Psychiatry 64, no. S1 (2021): S148, 10.1192/j.eurpsy.2021.405.

[28] H. Abboud , J. C. Probasco , S. Irani , et al., “Autoimmune Encephalitis: Proposed Best Practice Recommendations for Diagnosis and Acute Management,” Journal of Neurology, Neurosurgery, and Psychiatry 92, no. 7 (2021): 757–768, 10.1136/jnnp-2020-325300. PMC8223680 33649022

